# On MV-Algebraic Versions of the Strong Law of Large Numbers

**DOI:** 10.3390/e21070710

**Published:** 2019-07-19

**Authors:** Piotr Nowak, Olgierd Hryniewicz

**Affiliations:** Systems Research Institute, Polish Academy of Sciences, Newelska 6, 01-447 Warsaw, Poland

**Keywords:** Brunk–Prokhorov SLLN, Korchevsky SLLN, Marcinkiewicz–Zygmund SLLN, MV-algebraic probability

## Abstract

Many-valued (MV; the many-valued logics considered by Łukasiewicz)-algebras are algebraic systems that generalize Boolean algebras. The MV-algebraic probability theory involves the notions of the state and observable, which abstract the probability measure and the random variable, both considered in the Kolmogorov probability theory. Within the MV-algebraic probability theory, many important theorems (such as various versions of the central limit theorem or the individual ergodic theorem) have been recently studied and proven. In particular, the counterpart of the Kolmogorov strong law of large numbers (SLLN) for sequences of independent observables has been considered. In this paper, we prove generalized MV-algebraic versions of the SLLN, i.e., counterparts of the Marcinkiewicz–Zygmund and Brunk–Prokhorov SLLN for independent observables, as well as the Korchevsky SLLN, where the independence of observables is not assumed. To this end, we apply the classical probability theory and some measure-theoretic methods. We also analyze examples of applications of the proven theorems. Our results open new directions of development of the MV-algebraic probability theory. They can also be applied to the problem of entropy estimation.

## 1. Introduction

MV-algebras, being generalizations of Boolean algebras, were introduced by Chang [1] and used in the analysis of many-valued logic. Since this time, the theory of MV-algebras (see [2] and the references therein) has been considerably developed. The role of MV-algebras in the theory of quantum structures was discussed in [3,4].

Carathéodory defined the basic notions of point-free probability, replacing Kolmogorovian probability measures on σ-algebras by strictly positive probability measures on σ-complete Boolean algebras and random variables, defined within the Kolmogorov probability theory as measurable functions on the event space Ω, by functions from the σ-algebra of Borel subsets of R into the σ-Boolean algebra of events (see, e.g., [5]). The MV-algebraic probability theory generalizes the Boolean theory of probability, built by Carathéodory and von Neumann. In this paper, we assume that a state m:M→[0,1] on an MV-algebra *M* is a normalized σ-additive functional. Therefore, for each observable $x:B\left(R\right)\to M$, the function $m_{x}:B\left(R\right)\to \left[0,1\right]$ of the form $m_{x}\left(A\right)=m\left(x\left(A\right)\right)$, $A\in B\left(R\right)$, is a probability measure. Another notion of a state, which is not considered in this paper, was introduced by Mundici [6] as a normalized additive functional s:M→[0,1]. The σ-additivity of such a functional is obtained via the Kroupa–Panti theorem (see, e.g., Mundici [7]) and Riesz representation.

Main theorems of the MV-algebraic probability theory, including the basic version of the central limit theorem (CLT), laws of large numbers, and the individual ergodic theorem, can be found in [4,8,9]. In the MV-algebraic setting, there exist three versions of the SLLN (see [4,8]). The first one concerns the convergence of an independent sequence ${\left\{x_{i}\right\}}_{i\in N}$ of square-integrable observables in a probability MV-algebra $\left(M,m\right)$, satisfying the Kolmogorov condition (K): $\sum_{i=1}^{\infty }\frac{\sigma^{2}\left(x_{i}\right)}{i^{2}}<\infty ,$ where $\sigma^{2}\left(x_{i}\right)$ is the variance of $x_{i}$, i∈N. The analogous version of the SLLN was proven for a strongly independent sequence ${\left\{x_{i}\right\}}_{i\in N}$ of square-integrable weak observables, satisfying (K). The third MV-algebraic version of the strong law of large numbers concerns the convergence of a ⊙-independent sequence ${\left\{x_{i}\right\}}_{i\in N}$ of square-integrable weak observables, satisfying (K), under the additional assumption that the considered MV-algebra *M* is weakly σ-distributive. Generalized versions of the MV-algebraic central limit theorem, i.e., the Lindeberg and Lyapunov CLT, as well as the Feller theorem were proven by Nowak and Hryniewicz [10]. It is important to underline that, similar to in the Kolmogorov probability theory, the MV-algebraic versions of central limit theorem and strong law of large numbers are different types of theorems, since they concern different types of convergence of scaled sums of observables, i.e., the convergence in distribution in the first case and the convergence m-almost everywhere in the second case.

The MV-algebraic probability theory was also applied in the Atanassov intuitionistic fuzzy sets and interval-valued fuzzy sets settings (see, e.g., [11,12,13,14,15,16,17,18]). The notion of probability for the Atanassov intuitionistic fuzzy sets was introduced by Szmidt and Kacprzyk [19]. Some interesting aspects of the MV-algebraic probability theory were also studied in [20,21,22,23], etc.

As we have mentioned before, Riečan proved the MV-algebraic version of the SLLN for independent observables satisfying the Kolmogorov condition. This is a counterpart of the classical Kolmogorov theorem for independent square-integrable random variables, which is important for the Kolmogorov probability theory. However, in many practical problems, its assumptions are not satisfied, e.g., in the case where the second moments of the random variables do not exist or the random variables are dependent. In such situations, other strong laws of large numbers, including the Marcinkiewicz–Zygmund, Brunk–Prokhorov, and Korchevsky SLLN, are effective tools. In this paper, we prove generalized versions of the SLLN, i.e., the Marcinkiewicz–Zygmund, Brunk–Prokhorov, and Korchevsky SLLN, within the MV-algebraic probability theory, applying their classical counterparts and some measure-theoretic methods. The first two theorems, which were also proven by the authors in a non-MV-algebraic interval-valued fuzzy sets setting (see [24]), are devoted to sequences of independent observables in a probability MV-algebra, whereas the last one concerns observables taking values in a probability MV-algebra with the product, and their independence is not required. Since the above-mentioned classical versions of the SLLN are very useful from the theoretical point of view, we believe that their MV-algebraic counterparts will contribute to further development of the MV-algebraic probability theory, including the theory of stochastic processes in the MV-algebraic setting, and will be very useful for its applications to estimation methods.

We present three examples of the applications of the Marcinkiewicz–Zygmund, Brunk–Prokhorov, and Korchevsky SLLN for sequences of observables with convergent scaled sums. In particular, independent identically continuously distributed, as well as both independent and dependent discretely-distributed observables are considered.

The problem of entropy estimation is important from both theoretical and practical points of view. Classical versions of the law of large numbers are used in this field. In particular, the authors of [25,26,27] applied the SLLN to the analysis of the estimation of information theoretic measures, including entropy and Kullback–Leibler divergence. On the other hand, in [28], the concept of logical entropy was studied in the case of the family of IF-events, which can be embedded into a suitable MV-algebra. The results obtained in this paper are treated by us as tools to introduce methods of estimation of logical entropy, as well as other types of entropy in the case mentioned above.

The paper is organized as follows. Section 2 contains notations and selected elements of the MV-algebraic probability theory. The main results are presented in Section 3. The MV-algebraic versions of the Marcinkiewicz–Zygmund, Brunk–Prokhorov, and Korchevsky SLLN are proven there. Examples of applications of the SLLNs are discussed in Section 4. The paper is concluded in Section 5.

## 2. Preliminaries

We present some notations that are used in the paper.

We denote by R and N the set of real numbers and positive integers, respectively. For n∈N, the symbol $B(R^{n})$ denotes the σ-algebra of Borel subsets of $R^{n}$.

Let $\left(X,X\right)$ and $\left(X^{\prime },X^{\prime }\right)$ be two measurable spaces. A mapping $T:X\to X^{\prime }$ is measurable $X/X^{\prime }$ if $T^{-1}\left(A^{\prime }\right)\in X$ for each $A^{\prime }\in X^{\prime }$.

For each real-valued random variable *X* on a probability space $\left(\Omega ,S,P\right)$, we denote by EX the expected value of *X* and by ${E|X|}^{p}$ (for p>0) the absolute $p^{\mathrm{th}}$ moment of *X* with respect to *P* (if they exist).

### Selected Elements of the MV-Algebraic Probability Theory

The foundations of the theory of MV-algebras can be found in [2]. In this section, we recall only basic definitions and facts concerning MV-algebras and the MV-algebraic probability theory based on [4,10].

**Definition** **1.** *An MV-algebra* $\left(M,0,1,\neg ,⊕,⊙\right)$ *is an algebra, where M is a non-empty set, the operation *⊕* is associative and commutative with zero as the neutral element,* ¬0=1,¬1=0, *and for each* x,y∈M *:*

$$\begin{array}{c} x⊕1=1,\\ y⊕\neg \left(y⊕\neg x\right)=x⊕\neg \left(x⊕\neg y\right),\\ x⊙y=\neg \left(\neg x⊕\neg y\right). \end{array}$$

*In an MV-algebra* $\left(M,0,1,\neg ,⊕,⊙\right)$ *, a partial order is defined by the relation:*

x≤y⇔x⊙¬y=0,x,y∈M.

*The underlying lattice of M is the distributive lattice* $\left(M,\vee ,\wedge \right)$ *with the least element zero and the greatest element one, where the join and the meet are defined as follows:* $x\vee y=\neg \left(\neg x⊕y\right)⊕y;x\wedge y=\neg \left(\neg x\vee \neg y\right)$ *for each* x,y∈M *.*

**Definition** **2.** *An MV-algebra M is called σ-complete (complete) if every sequence (non-empty set, respectively) of elements of M has the supremum in M.*

For a non-empty set *X* and an MV-algebra *M*, we introduce the notations:

for ${\left\{A_{n}\right\}}_{n=1}^{\infty }\subset 2^{X}A_{n}↗A\;\mathrm{iff}\;A_{1}\subseteq A_{2}\subseteq \ldots \;\mathrm{and}\;{⋃}_{n}^{\infty }A_{n}=A,$
for ${\left\{x_{n}\right\}}_{n=1}^{\infty }\subset Rx_{n}↗x\;\mathrm{iff}\;x_{1}\leq x_{2}\leq \ldots \;\mathrm{and}\;x=\sup_{i}x_{i},$
for ${\left\{b_{n}\right\}}_{n=1}^{\infty }\subset Mb_{n}↗b\;\mathrm{iff}\;b_{1}\leq b_{2}\leq \ldots \;\mathrm{and}\;b=\sup_{i}b_{i}.$

**Definition** **3.** *Given a σ-complete MV-algebra M, a function* $m:M\to \left[0,1\right]$ *is called a state on M if it satisfies the following conditions for each* a,b,c∈M *and* ${\left\{a_{n}\right\}}_{n=1}^{\infty }\subset M$ *:*

*(i)*  $m\left(1\right)=1;$
*(ii)*  *if* b⊙c=0 *, then* $m\left(b⊕c\right)=m\left(b\right)+m\left(c\right);$
*(iii)*  *if* $a_{n}↗a$ *, then* $m\left(a_{n}\right)↗m\left(a\right).$

*A state m on M is called faithful if* $m\left(x\right)\neq 0$ *for each non-zero element x of M.*

**Definition** **4.** *A probability MV-algebra is a pair* $\left(M,m\right)$ *consisting of a σ-complete MV-algebra M and a faithful state m on M.*

If $\left(M,m\right)$ is a probability MV-algebra, then *M* is complete (see Theorem 13.8 in [7]).

**Definition** **5.** *Given a σ-complete MV-algebra M, a function* $x:B\left(R^{n}\right)\to M$ *is called an n-dimensional observable in M if it satisfies the following conditions:*

*(i)*  $x\left(R^{n}\right)=1;$
*(ii)*  $x\left(A\right)⊙x\left(B\right)=0$ *and* $x\left(A\cup B\right)=x\left(A\right)⊕x\left(B\right)$ *for each* $A,B\in B\left(R^{n}\right)$ *such that* A∩B=⌀;
*(iii)*  *for each* $A,A_{1},A_{2},\ldots \in B\left(R^{n}\right)\mathrm{if}\;A_{n}↗A,\;\mathrm{then}\;x\left(A_{n}\right)↗x\left(A\right).$

**Theorem** **1.** *Given M a σ-complete MV-algebra, an n-dimensional observable* $x:B\left(R^{n}\right)\to M$ *, and a state m on M, the function* $m_{x}:B\left(R^{n}\right)\to \left[0,1\right]$ *described by:*

$$m_{x}\left(A\right)=\left(m\circ x\right)\left(A\right)=m\left(x\left(A\right)\right),\;A\in B\left(R^{n}\right),$$

*is a probability measure on* $B\left(R^{n}\right)$ *.*

The proof of the above theorem can be found in [10].

**Definition** **6.** *Let* $\left(M,m\right)$ *be a probability MV-algebra. We call an observable* $x:B\left(R\right)\to M$ *integrable in* $\left(M,m\right)$ *if the expectation* $Ex=\int_{R}tdm_{x}\left(t\right)$ *exists. Moreover, we write* $x\in L_{m}^{p}$ *for* p>0 *if* ${E|x|}^{p}=\int_{R}{\left|t\right|}^{p}dm_{x}\left(t\right)<\infty ,$ *where* ${E|x|}^{p}$ *is the absolute* $p^{\mathrm{th}}$ *moment of x. If* $x\in L_{m}^{2}$ *, then the variance of x is given by the formula* $D^{2}x=Ex^{2}-{\left(Ex\right)}^{2}$ *.*

**Definition** **7.** *Observables* $x_{1},x_{2},$ …, $x_{n}$ *in a probability MV-algebra* $\left(M,m\right)$ *are said to be independent (with respect to m) if there exists an n-dimensional observable* $h:B\left(R^{n}\right)\to M$ *(called the joint observable of* $\;x_{1},x_{2},$ …, $x_{n}$ *) such that for arbitrary* $C_{1},C_{2},$ …, $C_{n}\in B\left(R\right)$ *:*

$$m\left(h\left(C_{1}\times C_{2}\times \ldots \times C_{n}\right)\right)=m_{x_{1}}\left(C_{1}\right)m_{x_{2}}\left(C_{2}\right)\ldots m_{x_{n}}\left(C_{n}\right).$$

**Remark** **1.** *Let* $x_{1},x_{2},\ldots ,x_{n}:B\left(R\right)\to M$ *be independent observables in a probability MV-algebra* $\left(M,m\right)$ *and* $h_{n}:B\left(R^{n}\right)\to M$ *be their joint observable. Then, for arbitrary Borel measurable function* $g_{n}:R^{n}\to R$ *, the mapping given by:*

(1) $$g_{n}\left(x_{1},x_{2},\ldots ,x_{n}\right)=h_{n}\circ g_{n}^{-1}$$

*is an observable.*

Convergence almost everywhere of observables in a probability MV-algebra was defined by Riečan and Mundici [4].

**Definition** **8.** *A sequence* ${\left\{z_{n}\right\}}_{n=1}^{\infty }$ *of observables in a probability MV-algebra* $\left(M,m\right)$ *is said to converge to zero m-almost everywhere (m-a.e.), if:*

(2) $$\lim_{p\to \infty }\lim_{k\to \infty }\lim_{i\to \infty }m\left(\overset{k+i}{\underset{n=k}{⋀}}z_{n}\left(\left(\frac{-1}{p},\frac{1}{p}\right)\right)\right)=1.$$

## 3. Generalized Versions of the MV-Algebraic Strong Law of Large Numbers

### 3.1. The Kolmogorov Probability Space of Observables

In the further part of this section, we will use the Kolmogorov probability space of observables considered by Riečan and Mundici [4].

Let ${\left\{x_{i}\right\}}_{i=1}^{\infty }$ be a sequence of independent observables in a probability MV-algebra $\left(M,m\right).$ Let $R^{N}$ be the space of all sequences of real numbers. Let C be the collection of cylinders of $R^{N}$, i.e., the collection of all sets $C=X_{i\in N}C_{i}\subset R^{N}$, where $C_{i}\in B\left(R\right)$ and $\left\{i:C_{i}\neq R\right\}$ is finite. The probability measure $P:B\left(R^{N}\right)\to \left[0,1\right]$ is uniquely described by:

$$P\left(C\right)=\prod_{i}m_{x_{i}}\left(C_{i}\right)$$

for every C∈C of the above form.

We call the triplet $\left(R^{N},B\left(R^{N}\right),P\right)$ the *Kolmogorov probability space of the observables* ${\left\{x_{i}\right\}}_{i=1}^{\infty }$ in $\left(M,m\right).$

For each n∈N, we define the $n^{\mathrm{th}}$ coordinate random variable ${ı}_{n}:R^{N}\to R$ and $n^{\mathrm{th}}$ coordinate random vector ${\bar{ı}}_{n}:R^{N}\to R^{n}$ by the formulas:

(3) $$\begin{array}{c} {ı}_{n}\left(u_{1},u_{2},\ldots \right)=u_{n},\\ {\bar{ı}}_{n}\left(u_{1},u_{2},\ldots \right)=\left(u_{1},u_{2},\ldots ,u_{n}\right). \end{array}$$

We recall a shortened version of Proposition 2.14 from [4].

**Proposition** **1.** *Let* $\left(M,m\right)$ *be a probability MV-algebra and* ${\left\{x_{i}\right\}}_{i=1}^{\infty }$ *be a sequence of independent observables in* $\left(M,m\right)$ *, with* $h_{n}:B\left(R^{n}\right)\to M$ *being the joint observable of* ${\left\{x_{i}\right\}}_{i=1}^{n}$ *. Let* $\left(R^{N},B\left(R^{N}\right),P\right)$ *be the Kolmogorov probability space of the observables* ${\left\{x_{i}\right\}}_{i=1}^{\infty }$ *in* $\left(M,m\right)$ *. For each* n∈N, *let* $g_{n}:R^{n}\to R$ *be an arbitrary Borel function. Let further the observable* $y_{n}:B\left(R\right)\to M$ *be defined by* $y_{n}=h_{n}\circ g_{n}^{-1}$ *and the random variable* $\eta_{n}:R^{N}\to R$ *be described by* $\eta_{n}=g_{n}\left({\bar{ı}}_{n}\right)$ *. Then:*

(4) $$P\circ \eta_{n}^{-1}=m\circ y_{n}$$

*and the convergence of* ${\left\{\eta_{i}\right\}}_{i=1}^{\infty }$ *to zero P-a.s.implies the convergence of* ${\left\{y_{i}\right\}}_{i=1}^{\infty }$ *to zero m-a.e.*

### 3.2. Generalized SLLN for Independent Observables

In this section, we formulate and prove MV-algebraic counterparts of the Marcinkiewicz–Zygmund and Brunk–Prokhorov SLLN (see Appendix A).

In the following part of the paper, in formulas containing integrals, we will assume that $t\in R^{k}$ for an appropriate value of k∈N.

Let $\left(X,X\right)$ and $\left(X^{\prime },X^{\prime }\right)$ be measurable spaces and a function $T:X\to X^{\prime }$ be $X/X^{\prime }$ measurable. For a given measure μ on X, the *image measure* $\mu T^{-1}$ on $X^{\prime }$ has the form:

$$\mu T^{-1}\left(A^{\prime }\right)=\mu \left(T^{-1}\left(A^{\prime }\right)\right),\;A^{\prime }\in X^{\prime }.$$

We will use the following lemma (a generalization of Lemma 3.2 from [10]), which shows the form of the expected value of a Borel function of an observable.

**Lemma** **1.** *Let* $\left(M,m\right)$ *be a probability MV-algebra and* d∈N *. Then, for any* R *-valued Borel function* $\varphi :R^{d}\to R$ *and observables* $h:B\left(R^{d}\right)\to M,\;y=\varphi \left(h\right):B\left(R\right)\to M$ *in* $\left(M,m\right)$ *, the expected value* $E\left(y\right)$ *exists if and only if:*

$$\int_{R^{d}}\left|\varphi \left(t\right)\right|dm_{h}\left(t\right)<\infty .$$

*Furthermore, if the above condition is satisfied, then:*

$$E\left(y\right)=\int_{R^{d}}\varphi \left(t\right)dm_{h}\left(t\right).$$

**Proof.**  Let $\left(X,X\right)$$=\left(R^{d},B\left(R^{d}\right)\right)$, $\left(X^{\prime },X^{\prime }\right)=\left(R,B\left(R\right)\right)$, and T=φ. By Theorem 1, $\mu =m_{h}$ is a probability measure. Furthermore, by straightforward computations, we obtain:

$$\mu T^{-1}=m_{\varphi \left(h\right)}=m_{y}.$$

Therefore, application of the following change of variable formula (see Theorem 16.12 in [29]):

(5) $$\int_{X}Tx\;d\mu \left(x\right)=\int_{X^{\prime }}x^{\prime }\;d\left(\mu T^{-1}\right)\left(x^{\prime }\right)$$

ends the proof. □

The MV-algebraic version of the Marcinkiewicz–Zygmund SLLN concerns the case of independent observables belonging to $L_{m}^{p}$ for $p\in \left(0,2\right)$ and a normalizing sequence that is a suitable power of *n* as follows.

**Theorem** **2.** *Given a probability MV-algebra* $\left(M,m\right)$ *, let* ${\left\{x_{i}\right\}}_{i=1}^{\infty }$ *be an independent sequence of observables in* $\left(M,m\right)$ *having the same distribution* $m_{x_{1}}$ *. Let* $p\in \left(0,2\right)$ *,* $x_{1}\in L_{m}^{p}$ *,* c=0 *for* 0<p<1 *and* $c=E\left(x_{1}\right)$ *for* 1≤p<2 *. Then:*

$$\frac{x_{1}+x_{2}+\ldots +x_{n}-nc}{n^{1/p}}$$

*converges to zero m-a.e.*

**Proof.**  Let $\left(R^{N},B\left(R^{N}\right),P\right)$ be the Kolmogorov probability space of the observables ${\left\{x_{i}\right\}}_{i=1}^{\infty }$ in $\left(M,m\right)$. The sequence ${\left\{{ı}_{i}\right\}}_{i=1}^{\infty }$ of the coordinate random variables is independent and identically distributed, i.e.,

$$P\circ {ı}_{i}^{-1}=m_{x_{i}}=m_{x_{1}}.$$

Thus, applying Lemma 1, we obtain:

$$E{ı}_{i}=E\left(x_{i}\right)=c\;\;\mathrm{for}\;1\leq p<2\;\;\mathrm{and}\;\;E{\left|{ı}_{i}\right|}^{p}=E\left(|x_{i}{|}^{p}\right)<\infty ,\;\;i\in N.$$

Then, c=0 for 0<p<1 and $c=E{ı}_{1}$ for 1≤p<2. For each n∈N, we denote by $h_{n}$ the joint observable of ${\left\{x_{i}\right\}}_{i=1}^{n}$, by $g_{n}$ the function:

$$g_{n}\left(t_{1},t_{2},\ldots ,t_{n}\right)=\frac{t_{1}+t_{2}+\ldots +t_{n}-nc}{n^{1/p}}$$

and by $y_{n}$ the observable:

$$y_{n}=h_{n}\circ g_{n}^{-1}.$$

We also introduce the sequence of random variables ${\left\{\eta_{n}\right\}}_{n=1}^{\infty }$, assuming that $\eta_{n}=g_{n}\left({\bar{ı}}_{n}\right)$. From Theorem A1, it follows that ${\left\{\eta_{n}\right\}}_{n=1}^{\infty }$ converges to zero a.s. By Proposition 1:

$$P\circ \eta_{n}^{-1}=m_{y_{n}}$$

and $y_{n}$ converges to zero *m*-a.e. □

The following MV-algebraic version of the Brunk–Prokhorov SLLN concerns sequences of observables that are not necessarily identically distributed.

**Theorem** **3.** *Let* p≥2 *. Given a probability MV-algebra* $\left(M,m\right)$ *, let* ${\left\{x_{i}\right\}}_{i=1}^{\infty }$ *be an independent sequence of observables in* $\left(M,m\right)$ *such that* $Ex_{i}=0$ *,* $x_{i}\in L_{m}^{p}$ *for each* i∈N *. Let:*

(6) $$\sum_{i=1}^{\infty }\frac{E|x_{i}{|}^{p}}{i^{p/2+1}}<\infty .$$

*Then:*

$$\lim_{n\to \infty }\frac{x_{1}+x_{2}+\ldots +x_{n}}{n}=0\;m\text{-}a.e.$$

**Proof.**  We denote by $\left(R^{N},B\left(R^{N}\right),P\right)$ the Kolmogorov probability space of the observables ${\left\{x_{i}\right\}}_{i=1}^{\infty }$ in $\left(M,m\right).$ For the independent sequence ${\left\{{ı}_{i}\right\}}_{i=1}^{\infty }$ of the coordinate random variables, by Lemma 1,

$$E{ı}_{i}=E\left(x_{i}\right)=0\;\;\mathrm{and}\;\;E|{ı}_{i}{|}^{p}=E{\left|x_{i}\right|}^{p}<\infty ,\;\;i\in N.$$

Therefore, by (6) and Theorem A2,

$$\lim_{n\to \infty }\frac{{ı}_{1}+{ı}_{2}+\ldots +{ı}_{n}}{n}=0\;\;P\text{-}a.s.$$

Thus, applying Proposition 1 for ${\left\{g_{n}\right\}}_{n=1}^{\infty }$ of the form:

$$g_{n}\left(t_{1},t_{2},\ldots ,t_{n}\right)=\frac{t_{1}+t_{2}+\ldots +t_{n}}{n},$$

we obtain the convergence:

$$\lim_{n\to \infty }\frac{x_{1}+x_{2}+\ldots +x_{n}}{n}=0\;\;m\text{-}a.e.$$

 □

### 3.3. Generalized SLLN for Non-Negative Observables

In this subsection, we consider the convergence of observables in a probability MV-algebra with product $\left(M,m,\cdot \right)$, i.e., a probability MV-algebra $\left(M,m\right)$ with an additional associative and commutative binary operation ·:M×M→M such that for each a,b,c∈M: (i)1·a=a; (ii) $a\cdot \left(b⊙\neg c\right)=\left(a\cdot b\right)⊙\neg \left(a\cdot c\right)$.

**Remark** **2.** *Let* $x_{1},x_{2},\ldots ,x_{n}:B\left(R\right)\to M$ *be observables in a probability MV-algebra with product* $\left(M,m,\cdot \right)$ *. By Proposition 2.4 from [4], each probability MV-algebra is weakly σ-distributive. Therefore, Theorem 3.6 from [4] implies that there exists an n-dimensional observable* $h_{n}:B\left(R^{n}\right)\to M$ *such that for arbitrary* $C_{1},C_{2},$ …, $C_{n}\in B\left(R\right)$ *:*

$$h_{n}\left(C_{1}\times C_{2}\times \ldots \times C_{n}\right)=x_{1}\left(C_{1}\right)\cdot x_{2}\left(C_{2}\right)\cdot \ldots \cdot x_{n}\left(C_{n}\right),$$

*called the joint observable of* $x_{1},x_{2},\ldots ,x_{n}$ *. Moreover, for arbitrary Borel measurable function* $g_{n}:R^{n}\to R$ *, the formula:*

$$g_{n}\left(x_{1},x_{2},\ldots ,x_{n}\right)=h_{n}\circ g_{n}^{-1}:B\left(R\right)\to M$$

*defines a one-dimensional observable.*

Let ${\left\{x_{i}\right\}}_{i=1}^{\infty }$ be a sequence of observables in a probability MV-algebra with product $\left(M,m,\cdot \right)$. Let for each n∈N$\;h_{n}:B\left(R^{n}\right)\to M$ be the joint observable of $x_{1},x_{2},\ldots ,x_{n}$, defined in the above remark. Then, analogous to the case of probability MV-algebra, the application of the Kolmogorov consistency theorem for probability measures $P_{n}=m\circ h_{n}$ implies the existence of exactly one probability measure $P:B\left(R^{N}\right)\to \left[0,1\right]$ such that for each n∈N and $A\in B(R^{n})$, $P\left({\bar{ı}}_{n}^{-1}\left(A\right)\right)=P_{n}\left(A\right)$, where ${\bar{ı}}_{n}$ is given by (3). We call the triplet $\left(R^{N},B\left(R^{N}\right),P\right)$ the *Kolmogorov probability space of the observables* ${\left\{x_{i}\right\}}_{i=1}^{\infty }$ in $\left(M,m,\cdot \right)$. Both ${ı}_{n}:R^{N}\to R$ and ${\bar{ı}}_{n}:R^{N}\to R^{n}$ are random variables on $\left(R^{N},B\left(R^{N}\right),P\right)$.

The following proposition is a consequence of Theorem 3.17 and Proposition 3.16 from [4].

**Proposition** **2.** *Let* $\left(M,m,\cdot \right)$ *be a probability MV-algebra with product and* ${\left\{x_{i}\right\}}_{i=1}^{\infty }$ *be a sequence of observables in* $\left(M,m,\cdot \right)$ *, with* $h_{n}:B\left(R^{n}\right)\to M$ *being the joint observable of* ${\left\{x_{i}\right\}}_{i=1}^{n}$ *. Let* $\left(R^{N},B\left(R^{N}\right),P\right)$ *be the Kolmogorov probability space of the observables* ${\left\{x_{i}\right\}}_{i=1}^{\infty }$ *in* $\left(M,m,\cdot \right)$ *. For each* n∈N, *let* $g_{n}:R^{n}\to R$ *be an arbitrary Borel function. Let further the observable* $y_{n}:B\left(R\right)\to M$ *be defined by* $y_{n}=h_{n}\circ g_{n}^{-1}$ *and the random variable* $\eta_{n}:R^{N}\to R$ *be described by* $\eta_{n}=g_{n}\left({\bar{ı}}_{n}\right)$ *. Then, the convergence of* ${\left\{\eta_{i}\right\}}_{i=1}^{\infty }$ *to zero P-a.s. implies the convergence of* ${\left\{y_{i}\right\}}_{i=1}^{\infty }$ *to zero m-a.e.*

We formulate and prove the main theorem of this subsection. For its classical counterpart and some notations, we refer the reader to Appendix A.

**Theorem** **4.** *Let observables* ${\left\{x_{i}\right\}}_{i=1}^{\infty }$ *in a probability MV-algebra with product* $\left(M,m,\cdot \right)$ *be non-negative, i.e.,*

$$m\left(x_{i}(\left[0,\infty \right)\right)=1,\;i\in N,$$

*and their absolute moments of some order* p≥1 *be finite. Let* ${\left\{a_{n}\right\}}_{n=1}^{\infty }$ *be a non-decreasing unbounded sequence of positive numbers. If* $\;Es_{n}=O\left(a_{n}\right)$ *, where:*

$$s_{n}=x_{1}+x_{2}+\ldots +x_{n},\;n\in N,$$

*and:*

$$E|x_{1}+x_{2}+\ldots +x_{n}-E\left(\sum_{i=1}^{n}x_{i}\right){|}^{p}=O\left(\frac{a_{n}^{p}}{\psi \left(a_{n}\right)}\right),$$

*where ψ is a function belonging to* $\Psi_{c}$ *, then:*

$$\lim_{n\to \infty }\frac{x_{1}+x_{2}+\ldots +x_{n}-\sum_{i=1}^{n}Ex_{i}}{a_{n}}=0\;m\text{-}a.e.$$

**Proof.**  Let $\left(R^{N},B\left(R^{N}\right),P\right)$ be the Kolmogorov probability space of the observables ${\left\{x_{i}\right\}}_{i=1}^{\infty }$ in $\left(M,m,\cdot \right)$. Let for arbitrary n∈N the functions $\varphi_{1,n},$$\varphi_{2,n}:R^{n}\to R$ be given by:

$$\begin{array}{c} \varphi_{1,n}\left(t_{1},t_{2},\ldots ,t_{n}\right)=t_{1}+t_{2}+\ldots +t_{n}\\ \varphi_{2,n}\left(t_{1},t_{2},\ldots ,t_{n}\right)=|t_{1}+t_{2}+\ldots +t_{n}-\sum_{i=1}^{n}Ex_{i}{|}^{p}, \end{array}$$

random variables $\eta_{1,n}$, $\eta_{2,n}$ have the forms

$$\eta_{1,n}=\varphi_{1,n}\left({\bar{ı}}_{n}\right),\;\;\eta_{2,n}=\varphi_{2,n}\left({\bar{ı}}_{n}\right)$$

and observables:

$$\begin{array}{c} s_{n}=\varphi_{1,n}\left(x_{1},x_{2},\ldots ,x_{n}\right):B\left(R\right)\to M,\\ y_{2,n}=\varphi_{2,n}\left(x_{1},x_{2},\ldots ,x_{n}\right):B\left(R\right)\to M \end{array}$$

be defined as in Remark 2. The distributions of coordinate random variables ${\left\{{ı}_{i}\right\}}_{i=1}^{\infty }$ are given by:

$$P\circ {ı}_{i}^{-1}=m_{x_{i}},\;i\in N.$$

Moreover,

$$P\circ {\bar{ı}}_{n}^{-1}=m_{h_{n}},\;n\in N.$$

Applying Lemma 1, we obtain:

$$E{ı}_{i}=E\left(x_{i}\right),\;\;E|{ı}_{i}{|}^{p}=E{\left|x_{i}\right|}^{p}<\infty ,\;\;i\in N,$$

and:

$$\begin{array}{c} E\eta_{1,n}=\int_{R^{N}}\varphi_{1,n}\left({\bar{ı}}_{n}\right)dP=\int_{R^{n}}\varphi_{1,n}\left(t\right)d\left(P\circ {\bar{ı}}_{n}^{-1}\right)\left(t\right)=\int_{R^{n}}\varphi_{1,n}\left(t\right)dm_{h_{n}}\left(t\right)\\ \;=Es_{n}=O\left(a_{n}\right),\\ E\eta_{2,n}=\int_{R^{N}}\varphi_{2,n}\left({\bar{ı}}_{i}\right)dP=\int_{R^{n}}\varphi_{2,n}\left(t\right)d\left(P\circ {\bar{ı}}_{i}^{-1}\right)\left(t\right)\\ \;=\int_{R^{n}}\varphi_{2,n}\left(t\right)dm_{h_{n}}\left(t\right)=Ey_{2,n}=O\left(\frac{a_{n}^{p}}{\psi \left(a_{n}\right)}\right),\;\;n\in N. \end{array}$$

Clearly, $P\left({ı}_{i}\in \left[0,\infty \right)\right)=m_{x_{i}}\left([0,\infty )\right)=1,\;i\in N.$ Finally, application of Theorem A3 for the sequence of random variables ${\left\{{ı}_{i}\right\}}_{i=1}^{\infty }$ and Proposition 2 for the sequence of Borel functions:

$$\begin{array}{c} g_{n}:R^{n}\to R,\;n\in N,\\ g_{n}\left(t_{1},t_{2},\ldots ,t_{n}\right)=\frac{t_{1}+t_{2}+\ldots +t_{n}-\sum_{i=1}^{n}Ex_{i}}{a_{n}} \end{array}$$

ends the proof. □

We also present two special cases of the above theorem, corresponding to Theorem 2 and 3, formulated by Korchevsky [30] within the Kolmogorov probability theory.

**Theorem** **5.** *Let observables* ${\left\{x_{i}\right\}}_{i=1}^{\infty }$ *in a probability MV-algebra with product* $\left(M,m,\cdot \right)$ *be non-negative and have finite variances. Let* ${\left\{a_{n}\right\}}_{n=1}^{\infty }$ *be a non-decreasing unbounded sequence of positive numbers. If* $\;Es_{n}=O\left(a_{n}\right)$ *and:*

$$D^{2}s_{n}=O\left(\frac{a_{n}^{2}}{\psi \left(a_{n}\right)}\right)$$

*for some function* $\psi \in \Psi_{c}$ *, then:*

$$\lim_{n\to \infty }\frac{x_{1}+x_{2}+\ldots +x_{n}-\sum_{i=1}^{n}Ex_{i}}{a_{n}}=0\;m\text{-}a.e.$$

**Theorem** **6.** *Let observables* ${\left\{x_{i}\right\}}_{i=1}^{\infty }$ *in a probability MV-algebra with product* $\left(M,m,\cdot \right)$ *be non-negative and have finite absolute moments of some order* p≥1 *. Let* ${\left\{w_{n}\right\}}_{n=1}^{\infty }$ *be a sequence of positive numbers,*

$$W_{n}=\sum_{i=1}^{n}w_{i},\;\;\tau_{n}=\sum_{i=1}^{n}w_{i}x_{i},\;\;n\in N,$$

$\lim_{n\to \infty }W_{n}=\infty$ *,* $E\tau_{n}=O\left(W_{n}\right)$ *and:*

$$E|\tau_{n}-E\tau_{n}{|}^{p}=O\left(\frac{W_{n}^{p}}{\psi \left(W_{n}\right)}\right)$$

*for some function* $\psi \in \Psi_{c}$ *. Then:*

$$\lim_{n\to \infty }\frac{\tau_{n}-E\tau_{n}}{W_{n}}=0\;m\text{-}a.e.$$

Theorem 5 follows from Theorem 4 applied for p=2, whereas Theorem 6 is a consequence of Theorem 4 for arbitrary p≥1, the sequence of observables $y_{i}=w_{i}x_{i}$, n∈N, and the sequence of real numbers $a_{n}=W_{n}$, n∈N.

## 4. Illustrative Examples

We analyze the asymptotic behavior of scaled sums of three sequences of observables. They are independent identically continuously distributed in the first sequence and independent identically discretely distributed in the second one, whereas the third example concerns non-negative dependent observables. The MV-algebraic version of the Kolmogorov SLLN, presented in [9], cannot be applied in the first two examples. Therefore, we use the MV-algebraic versions of the Marcinkiewicz–Zygmund and Brunk–Prokhorov theorems. The case of dependent observables is new, and it requires application of the MV-algebraic version of the Korchevsky theorem.

### 4.1. Sequence of Identically Distributed Observables

We consider observables taking values in the probability MV-algebra $\left(M,m\right)$ consisting of the MV-algebra $M=\left[0,1\right]$ equipped with the operations ¬,⊕,⊙ defined by the formulas:

$$\neg a=1-a,\;a⊕b=\left(a+b\right)\wedge 1,\;a⊙b=\left(a+b-1\right)\vee 0,$$

where +, −, ∧, and ∨ have the usual meaning of addition, subtraction, minimum, and maximum, and the faithful state *m* of the form $m\left(t\right)=t$.

Let p>0, $\gamma =\frac{7}{4},$ $C_{\gamma }=\frac{3}{8}$, and:

$$f\left(t\right)=\frac{C_{\gamma }}{{\left|t\right|}^{\gamma }}I_{|t|\geq 1}\left(t\right).$$

Let for each j∈N an observable $x_{j}:B\left(R\right)\to M$ be described by the equality:

$$x_{j}\left(A\right)=\int_{A}f\left(t\right)dt.$$

We assume that the observables defined above are independent. For each n∈N, the joint observable $h_{n}:B\left(R^{n}\right)\to M$ of ${\left\{x_{j}\right\}}_{j=1}^{n}$ has the form of the product measure given for each $A\in B\left(R^{n}\right)$ by:

$$h_{n}\left(A\right)=\int_{A}f\left(t_{1}\right)f\left(t_{2}\right)\ldots f\left(t_{n}\right)dt_{1}dt_{2}\ldots dt_{n}.$$

Then, applying Lemma 1, we obtain:

$$E\left(|x_{1}{|}^{p}\right)=2C_{\gamma }\int_{1}^{\infty }t^{p-\frac{7}{4}}dt=\left\{\begin{array}{ccc} \frac{2C_{\gamma }}{\gamma -1-p}<\infty & for & p<\gamma -1;\\ \infty & for & p\geq \gamma -1. \end{array}\right.$$

Thus, $E\left(x_{1}^{2}\right)=\infty$, and therefore, the MV-algebraic version of the Kolmogorov SLLN, presented in [9], does not concern the considered case. However, application of Theorem 2 gives the convergence:

$$\frac{x_{1}+x_{2}+\ldots +x_{n}}{n^{r}}\vec{{}_{n\to \infty }}0\;\;m\text{-}a.e.$$

for arbitrary $r>\frac{1}{\gamma -1}=\frac{4}{3}.$

### 4.2. Sequence of Not Identically Distributed Observables

Let $\left(M,m\right)$ be the probability MV-algebra, defined in the previous subsection.

We fix p≥2. Let $e_{1}^{j}=-j^{1/4},$ $e_{2}^{j}=j^{1/4},$ $p_{1}^{j}=p_{2}^{j}=\frac{1}{2}$ for j∈N. Let a sequence of independent observables:

$$x_{j}:B\left(R\right)\to M,\;j\in N$$

be defined by for each $A\in B\left(R\right)$ by the equality:

$$x_{j}\left(A\right)=\sum_{e_{i}^{j}\in A,\;i\in \left\{1,2\right\}}p_{i}^{j}.$$

For an arbitrary positive integer *n*, the joint observable $h_{n}:B\left(R^{n}\right)\to M$ of the sequence ${\left\{x_{j}\right\}}_{j=1}^{n}$ is the product measure given for each $A\in B\left(R^{n}\right)$ by the equality:

$$\begin{array}{c} h_{n}\left(A\right)=\sum_{\left(e_{i_{1}}^{1},e_{i_{2}}^{2},\ldots ,e_{i_{{}_{n}}}^{n}\right)\in A,\;\;i_{1},i_{2},\ldots ,i_{n}\in \left\{1,2\right\}}p_{i_{1}}^{1}p_{i_{2}}^{2}\ldots p_{i_{n}}^{n}\\ \;=\sum_{\left(e_{i_{1}}^{1},e_{i_{2}}^{2},\ldots ,e_{i_{{}_{n}}}^{n}\right)\in A,\;\;i_{1},i_{2},\ldots ,i_{n}\in \left\{1,2\right\}}\frac{1}{2^{n}}. \end{array}$$

Then, $E|x_{j}{|}^{p}=j^{p/4}<\infty$ for j∈N, and therefore,

$$\sum_{j=1}^{\infty }\frac{E|x_{j}{|}^{p}}{j^{p/2+1}}=\sum_{j=1}^{\infty }\frac{1}{j^{p/4+1}}<\infty .$$

Thus, by Theorem 3,

$$\frac{x_{1}+x_{2}+\ldots +x_{n}}{n}\vec{{}_{n\to \infty }}0\;\;m\text{-}a.e.$$

### 4.3. Sequence of Dependent Observables

We consider a probability MV-algebra with the product, presented in [4]. Let $\left(\Omega ,S,P\right)$ be a probability space and $F_{0}$ be the set of all *S*-measurable functions $f:\Omega \to \left[0,1\right]$. $F_{0}$, called a *full tribe*, equipped with the operations ¬,⊕,⊙,· given by:

$$\begin{array}{c} \;\left(\neg f\right)\left(x\right)=1-f\left(x\right),\;\left(f⊕g\right)\left(x\right)=\left(f\left(x\right)+g\left(x\right)\right)\wedge 1,\;\\ \left(f⊙g\right)\left(x\right)=\left(f\left(x\right)+g\left(x\right)-1\right)\vee 0,\left(f\cdot g\right)\left(x\right)=f\left(x\right)\cdot g\left(x\right),\;x\in \Omega , \end{array}$$

is an MV-algebra with product. $0=0_{\Omega }$ and $1=1_{\Omega },$ where for arbitrary $c\in \left[0,1\right]$ $c_{\Omega }$ is the function identically equal to *c* on Ω. Let a state $m_{0}$ on $F_{0}$ be defined by the formula:

$$m_{0}\left(f\right)=\int_{\Omega }fdP.$$

The ideal $J=\left\{f\in F_{0}:m_{0}\left(f\right)=0\right\}$ in $F_{0}$ is closed under countable suprema. Therefore, the quotient MV-algebra $F=F_{0}/J$ (with the operations ¬,⊕,⊙,· arising via θ) is σ-complete, the quotient map $\theta :F_{0}\to F$ is a σ-homomorphism, $m:F\to \left[0,1\right]$ given by $m\left(\theta \left(f\right)\right)=m_{0}\left(f\right)$ is a faithful state on F, and $\left(F,m,\cdot \right)$ is a probability MV-algebra with product (see [4]).

Let $\left(\Omega ,S,P\right)=\left(\left[0,1\right],B\left(\left[0,1\right]\right),\lambda_{\left[0,1\right]}\right)$, where $\lambda_{\left[0,1\right]}$ is the Lebesgue measure on $\left[0,1\right]$. For the fixed constant $\gamma =\frac{1}{2}$ and sequence ${\left\{\alpha_{i}\right\}}_{i=1}^{\infty }$, $\;\alpha_{i}=\frac{1}{2^{i}}$, we define a sequence ${\left\{z_{i}\right\}}_{i=1}^{\infty }$ of independent observables in $\left(F,m,\cdot \right)$, given for arbitrary $A\in B\left(R\right)$ by the equality:

$$z_{i}\left(A\right)=\left\{\begin{array}{ccc} \theta \left(1_{\Omega }\right) & \mathrm{if} & A\cap \left\{0,1\right\}=\left\{0,1\right\};\\ \theta \left(\gamma_{\Omega }\right) & \mathrm{if} & A\cap \left\{1\right\}=\left\{1\right\};\\ \theta \left({\left(1-\gamma \right)}_{\Omega }\right) & \mathrm{if} & A\cap \left\{0\right\}=\left\{0\right\};\\ \theta \left(0_{\Omega }\right) & \mathrm{if} & A\cap \left\{0,1\right\}=\emptyset \end{array}\right.$$

and a sequence ${\left\{x_{i}\right\}}_{i=1}^{\infty }$ of observables in $\left(F,m,\cdot \right)$ of the form:

$$x_{i}=\alpha_{i}z_{1}+\left(1-\alpha_{i}\right)z_{i},\;\;i\in N.$$

Let for n∈N $h_{n}$ be the joint observable of ${\left\{z_{i}\right\}}_{i=1}^{n},$

$$A_{n}=\sum_{i=2}^{n}\alpha_{i}=\frac{1}{2}-{\left(\frac{1}{2}\right)}^{n},\;B_{n}=\sum_{i=2}^{n}\alpha_{i}^{2}=\frac{1}{12}-\frac{1}{3}{\left(\frac{1}{4}\right)}^{n},\;a_{n}=n.$$

Clearly,

$$m_{z_{i}}\left(dt\right)=\left(\gamma \delta_{\left\{1\right\}}+\left(1-\gamma \right)\delta_{\left\{0\right\}}\right)\left(dt\right).$$

Let p≥1. Then, for $i\in NE|z_{i}{|}^{p}=Ez_{i}^{p}=\gamma ,\;D^{2}z_{i}=\gamma \left(1-\gamma \right),\;E{\left|x_{1}\right|}^{2}=\gamma$. Moreover, by Lemma 1, for i≥2:

$$\begin{array}{c} E|x_{i}{|}^{2}=E|\alpha_{i}z_{1}+\left(1-\alpha_{i}\right)z_{i}{|}^{2}=\int_{R^{i}}{\left|\alpha_{i}t_{1}+\left(1-\alpha_{i}\right)t_{i}\right|}^{2}dm_{h_{i}}\left(t\right)\\ \;=\int_{R^{2}}{\left(\alpha_{i}t_{1}+\left(1-\alpha_{i}\right)t_{i}\right)}^{2}dm_{z_{1}}\left(t_{1}\right)dm_{z_{i}}\left(t_{i}\right)\\ \;=\alpha_{i}^{2}\gamma +2\alpha_{i}\left(1-\alpha_{i}\right)\gamma^{2}+{\left(1-\alpha_{i}\right)}^{2}\gamma <\infty . \end{array}$$

Let for a fixed n∈N $\beta_{1}=1+A_{n}$ and $\beta_{i}=1-\alpha_{i}$, 2≤i≤n. Then, $s_{n}=\sum_{i=1}^{n}\beta_{i}z_{i}$, and by Lemma 1:

$$\begin{array}{c} Es_{n}=\int_{R^{n}}\left(\sum_{i=1}^{n}\beta_{i}t_{i}\right)dm_{h_{n}}\left(t\right)=\sum_{i=1}^{n}\int_{R}\beta_{i}t_{i}dm_{z_{i}}\left(t_{i}\right)=\sum_{i=1}^{n}\beta_{i}Ez_{i}\\ \;=\gamma \sum_{i=1}^{n}\beta_{i}=\gamma \left(1+A_{n}+n-1-A_{n}\right)=\gamma n=\frac{1}{2}n=O\left(a_{n}\right) \end{array}$$

as well as:

$$\begin{array}{c} E|s_{n}-Es_{n}{|}^{2}=E{\left(\sum_{i=1}^{n}\beta_{i}z_{i}-\sum_{i=1}^{n}\beta_{i}Ez_{i}\right)}^{2}\\ =\int_{R^{n}}{\left(\sum_{i=1}^{n}\beta_{i}t_{i}-\sum_{i=1}^{n}\beta_{i}Ez_{i}\right)}^{2}dm_{h_{n}}\left(t\right)=\int_{R^{n}}{\left(\sum_{i=1}^{n}\beta_{i}\left(t_{i}-Ez_{i}\right)\right)}^{2}dm_{h_{n}}\left(t\right)\\ =\int_{R^{n}}\left(\sum_{i=1}^{n}\beta_{i}^{2}{\left(t_{i}-Ez_{i}\right)}^{2}+2\sum_{1\leq i<j\leq n}\beta_{i}\beta_{j}\left(t_{i}-Ez_{i}\right)\left(t_{j}-Ez_{j}\right)\right)dm_{h_{n}}\left(t\right)\\ =\sum_{i=1}^{n}\beta_{i}^{2}\int_{R}{\left(t_{i}-Ez_{i}\right)}^{2}dm_{z_{i}}\left(t_{i}\right)\\ +2\sum_{1\leq i<j\leq n}\beta_{i}\beta_{j}\int_{R^{2}}\left(t_{i}-Ez_{i}\right)\left(t_{j}-Ez_{j}\right)dm_{z_{i}}\left(t_{i}\right)dm_{z_{j}}\left(t_{j}\right)=\sum_{i=1}^{n}\beta_{i}^{2}D^{2}z_{i}\\ =\gamma \left(1-\gamma \right)\sum_{i=1}^{n}\beta_{i}^{2}=\gamma \left(1-\gamma \right)\left[1+2A_{n}+A_{n}^{2}+n-1-2A_{n}+B_{n}\right]\\ =\frac{1}{4}\left[n+\frac{2}{3}{\left(\frac{1}{4}\right)}^{n}-{\left(\frac{1}{2}\right)}^{n}+\frac{1}{3}\right]=O\left(\frac{a_{n}^{2}}{\psi \left(a_{n}\right)}\right), \end{array}$$

where $\psi \left(x\right)=x\in \Psi_{c}$. Thus, by Theorem 5 (or Theorem 4 for p=2):

$$\lim_{n\to \infty }\frac{x_{1}+x_{2}+\ldots +x_{n}-n\gamma }{n}=0\;m\text{-}a.e.$$

## 5. Conclusions

This paper is devoted to the development of MV-algebraic probability theory. We formulate and prove three generalized versions of the strong law of large numbers. The first two versions of the strong law, i.e., the MV-algebraic Marcinkiewicz–Zygmund SLLN and Brunk–Prokhorov SLLN, describe the asymptotic behavior of the sums of independent observables, whereas the third one, i.e., the Korchevsky SLLN, concerns the case of dependent observables. Their proofs require an application of the Kolmogorov theory of probability and some measure-theoretic techniques. To illustrate our theoretical results, we also present and analyze some examples of sequences of observables in a probability MV-algebra. We believe that our results open new possibilities for further development of the MV-algebraic probability theory in the non-Kolmogorovian setting. In particular, they can be used for the future development of the theory of fuzzy, intuitionistic fuzzy, and interval-valued fuzzy random events in complex spaces. We would like to apply the proven theorems to the estimation of logical entropy, as well as other types of entropy in the case of intuitionistic fuzzy random events. This requires, among other things, the definition of entropy for observables in MV-algebras.

## Appendix A. Classical Versions of the SLLN

Let ${\left\{X_{i}\right\}}_{i=1}^{\infty }$ be a sequence of independent real-valued random variables on a probability space $\left(\Omega ,S,P\right)$, and let $S_{n}=X_{1}+X_{2}+\ldots +X_{n}$ for each n∈N. Apart from the Kolmogorov SLLN for identically distributed sequence ${\left\{X_{i}\right\}}_{i=1}^{\infty }$ such that $E|X_{1}|<\infty$, its Marcinkiewicz–Zygmund generalization is known (see [31]):

**Theorem** **A1** (Marcinkiewicz–Zygmund SLLN)**.** *Let the random variables* ${\left\{X_{i}\right\}}_{i=1}^{\infty }$ *be independent identically distributed. Let* $p\in \left(0,2\right)$*. If* $E|X_{1}{|}^{p}<\infty$*, then:*

$$\lim_{n\to \infty }\frac{S_{n}-nc}{n^{1/p}}=0\;P\text{-}a.s.$$

*In the above formula,* c=0 *if* 0<p<1 *and* $c=EX_{1}$ *if* 1≤p<2*.*

In the next theorem, the same distribution of the random variables is not required.

**Theorem** **A2** (Brunk–Prokhorov SLLN)**.** *Let the random variables* ${\left\{X_{i}\right\}}_{i=1}^{\infty }$ *be independent. Let* q≥1*. If* $EX_{i}=0$*,* $E|X_{i}{|}^{2q}<\infty$ *for each* i∈N *and:*

$$\sum_{i=1}^{\infty }\frac{E|X_{i}{|}^{2q}}{i^{q+1}}<\infty ,$$

*then* $\lim_{n\to \infty }\frac{S_{n}}{n}=0$ *P-a.s.*

The above theorem was proven by Brunk [32] for arbitrary positive integer *q* and by Prokhorov [33] for arbitrary q≥1.

Let $\Psi_{c}$ be the set of positive functions ψ(x), non-decreasing in the interval $x>x_{0}$ for some $x_{0}$, such that $\sum_{n=1}^{\infty }\frac{1}{n\psi (n)}<\infty$.

The following Korchevsky generalization of the Petrov SLLN (see [30]) gives a sufficient condition for the convergence of scaled sums of random variables without the independence condition.

**Theorem** **A3** (Korchevsky)**.** *Let the random variables* ${\left\{X_{i}\right\}}_{i=1}^{\infty }$ *be non-negative with finite absolute moments of some order* p≥1*. Let* ${\left\{a_{n}\right\}}_{n=1}^{\infty }$ *be a non-decreasing unbounded sequence of positive numbers. If* ${\mathrm{ES}}_{n}=O\left(a_{n}\right)$ *and there exists a function* $\psi \in \Psi_{c}$ *such that* $E|S_{n}-{\mathrm{ES}}_{n}{|}^{p}=O\left(\frac{a_{n}^{p}}{\psi \left(a_{n}\right)}\right)$*, then:*

$$\lim_{n\to \infty }\frac{S_{n}-{\mathrm{ES}}_{n}}{n}=0\;P\text{-}a.s.$$
